# Respiratory syncytial virus (RSV) entry is inhibited by serine protease inhibitor AEBSF when present during an early stage of infection

**DOI:** 10.1186/s12985-017-0824-3

**Published:** 2017-08-17

**Authors:** Winke Van der Gucht, Annelies Leemans, Marjorie De Schryver, Annick Heykers, Guy Caljon, Louis Maes, Paul Cos, Peter L. Delputte

**Affiliations:** Laboratory of Microbiology, Parasitology and Hygiene, University of Antwerp, Antwerp, Belgium

**Keywords:** RSV, Protease inhibitor, Host protease, AEBSF

## Abstract

**Background:**

Host proteases have been shown to play important roles in many viral activities such as entry, uncoating, viral protein production and disease induction. Therefore, these cellular proteases are putative targets for the development of antivirals that inhibit their activity. Host proteases have been described to play essential roles in Ebola, HCV, HIV and influenza, such that specific protease inhibitors are able to reduce infection. RSV utilizes a host protease in its replication cycle but its potential as antiviral target is unknown. Therefore, we evaluated the effect of protease inhibitors on RSV infection.

**Methods:**

To measure the sensitivity of RSV infection to protease inhibitors, cells were infected with RSV and incubated for 18 h in the presence or absence of the inhibitors. Cells were fixed, stained and studied using fluorescence microscopy.

**Results:**

Several protease inhibitors, representing different classes of proteases (AEBSF, Pepstatin A, E-64, TPCK, PMSF and aprotinin), were tested for inhibitory effects on an RSV A2 infection of HEp-2 cells. Different treatment durations, ranging from 1 h prior to inoculation and continuing for 18 h during the assay, were evaluated. Of all the inhibitors tested, AEBSF and TPCK significantly decreased RSV infection. To ascertain that the observed effect of AEBSF was not a specific feature related to HEp-2 cells, A549 and BEAS-2B cells were also used. Similar to HEp-2, an almost complete block in the number of RSV infected cells after 18 h of incubation was observed and the effect was dose-dependent. To gain insight into the mechanism of this inhibition, AEBSF treatment was applied during different phases of an infection cycle (pre-, peri- and post-inoculation treatment). The results from these experiments indicate that AEBSF is mainly active during the early entry phase of RSV. The inhibitory effect was also observed with other RSV isolates A1998/3–2 and A2000/3–4, suggesting that this is a general feature of RSV.

**Conclusion:**

RSV infection can be inhibited by broad serine protease inhibitors, AEBSF and TPCK. We confirmed that AEBSF inhibition is independent of the cell line used or RSV strain. The time point at which treatment with the inhibitor was most potent, was found to coincide with the expected moment of entry of the virion with the host cell.

## Background

Human respiratory syncytial virus (RSV) is the principal viral cause of serious lower respiratory tract infection (LRTI) in infants and young children, immunocompromised and cardiopulmonary diseased patients and also elderly [1]. RSV causes common cold like symptoms that can progress to LRTI leading to hospitalization, significant morbidity and even mortality [1–7]. By the age of three, nearly all children have been exposed to RSV at least once and re-infection is common throughout life [3, 8]. RSV has been estimated to cause 3.4 million LRTI episodes that require hospitalization and up to 199,000 deaths of children under 5 years old, mostly in developing countries [9]. No vaccines or therapeutics are currently on the market since the discovery of the virus in 1957 [10]. Only high-risk premature infants are qualified to receive expensive passive immunization of Synagis® (Palivizumab), a humanized monoclonal antibody that targets a conserved epitope of the RSV fusion protein [11]. At this time, treatment of severe RSV disease consists of supportive care, such as hydration and oxygenation.

RSV belongs to the *Pneumoviridae,* genus *Ortho* *pneumovirus,* which is comprised of enveloped viruses with a negative-stranded RNA genome. The 15.2 kb genome is non-segmented, single stranded and encodes 11 proteins in 10 genes [3]. Three of the proteins are present in the envelope of the virus: the attachment glycoprotein (G), the fusion glycoprotein (F) and the small hydrophobic (SH) protein. RSV entry has been theorized to follow the entry model of other paramyxoviruses, where the G protein initiates binding to the host cell through interactions with GAGs [12, 13], followed by F-mediated fusion of the host membrane with the viral envelop, allowing virus entry [14]. The location of RSV fusion has to be further elucidated, since evidence is available for RSV using different pathways into the cell. Main theories consist of fusion at the cell surface or entry by endocytic mechanisms such as macropinocytosis [15], caveolae [14, 16] or endosomes [14] followed by fusion.

Currently, neither vaccines or antiviral therapies against RSV have been approved and are available commercially. Therefore, other paths are being researched to discover alternative antiviral pathways and inhibition methods. In this view, host proteases which have been shown to be involved in many viral activities such as uncoating, viral protein production and post-translational modifications, provide potential antiviral targets through the use of protease inhibitors. The advantage of developing inhibitors for host proteins is that they generally have a reduced risk for the induction of drug resistance [17, 18]. For viruses such as Ebola [19], HCV, HIV [20], Influenza [21] and MERS [22], host proteases have been described that play an essential role in virus replication, allowing the use of specific protease inhibitors to reduce the infection.

RSV as well has been reported to utilize host proteases in its replication cycle. The RSV F protein is synthesized in the host cell as a 68 kDa precursor, F_0_, which is transported to the cell surface through the *trans-*Golgi network where it is activated by a double cleavage performed by furin [23], resulting in two disulfide-linked membrane anchored subunits of approximately 48 kDa (F_1_) and 20 kDa (F_2_) [24–26] and the release of a 27 amino acid long peptide called Pep27 [27]. This enzymatic processing is associated with structural changes and is required to activate the fusogenicity of the F protein [15, 28]. The RSV G protein was recently shown to be subjected to cleavage by Cathepsin L during endocytic recycling in Vero cells, resulting in a smaller size, but this feature seems to occur only in Vero cells [29].

Undoubtedly, more processes of the RSV replication cycle are in need of enzymatic modifications regulated by cellular host proteases, which may provide a new level of control on the RSV infection, while leaving the host cellular metabolism undisturbed through the use of redundant pathways.

In this study, we analyzed the RSV infection during the first replication cycle to quantify inhibitor effects in early replication phases. This allowed us to characterize the effects of several broad protease inhibitors on the early infection and replication of the RSV virus, thereby demonstrating that the serine protease inhibitor 4-(2-Aminoethyl) benzene sulfonyl fluoride hydrochloride (AEBSF) had a strong inhibitory effect on the infection. This inhibitory effect was present when AEBSF was administered during and after inoculation in HEp-2 cells. Not only did we observe this effect on laboratory strain RSV A2, but as well in clinical isolates and other immortalized cell lines.

## Methods

### Cells and viruses

The HEp-2 and A549 cell lines were obtained from ATCC. The BEAS-2B cells were a gift from dr. Ultan F. Power. Cells were grown in Dulbecco’s modified Eagle medium (DMEM) supplemented with 10% inactivated fetal bovine serum (iFBS) (Life technologies). RSV reference strain A2, and clinical isolates A1998/3–2 and A2000/3–4 were obtained from BEI resources and propagated in HEp-2 cells. Briefly, virus was added to a 70% confluent HEp-2 cell culture in a small volume of DMEM and left to adhere for 2 h on 37 °C (5% CO_2_). Afterwards, DMEM and iFBS were added to obtain a final concentration of 2% iFBS. The culture was left to grow for 2–3 days until cytopathic effects (CPE) were observed. Then the supernatant was collected, cleared by centrifugation (10′, 1000×g), aliquoted and snap frozen in liquid nitrogen. Plaque forming units (PFU) were determined in a conventional plaque assay on HEp-2 as described by Schepens B. et al. [30].

### Protease inhibitors (Table1)

All protease inhibitors were commercially available and purchased from Sigma-Aldrich. Working concentrations differed for each inhibitor.

4-(2-Aminoethyl) benzene sulfonyl fluoride hydrochloride (AEBSF) was dissolved at a concentration of 100 mM in DMEM. Working concentrations lay between 3 mM and 0,1 mM. Pepstatin A (PepA) was dissolved in ethanol at a concentration of 10 mM. Working concentrations were between 30 μM and 1 μM. Trans-epoxysuccinyl-L-leucylamido (4-guanido) butane (E-64) was dissolved in DMEM at a concentration of 1 mM. Working concentrations were kept between 30 μM and 1 μM. Aprotinin was dissolved at a concentration of 100 μM in DMEM. Working concentrations ranged from 2.4 μM to 0.08 μM. Phenyl methyl sulfonyl fluoride (PMSF) was dissolved at a concentration of 0.2 M in ethanol and used in experiments between 3 mM and 0.1 mM. Tosyl phenylalanyl chloromethyl ketone (TPCK) was dissolved in DMSO at 0.1 M. Working concentrations were 0.1 mM to 0.003 mM.

### Single infection cycle

At 24 h prior to infection, HEp-2 cells, A549 cells and BEAS2B cells were seeded at a concentration of 200,000 cells/ml in black cellstar® 96 well plates with a μclear® flat bottom suitable for fluorescence microscopy (Greiner Bio-one). Cells were briefly washed with DMEM without iFBS. DMEM was removed, replaced with virus inoculum (m.o.i. 1), diluted in DMEM without iFBS and left to adhere for 2 h on 37 °C (5% CO2). Afterwards, the inoculum was replaced with DMEM with 10% iFBS. The cells were incubated at 37 °C for 16 h, washed with PBS containing additional Ca^2+^ and Mg^2+^, fixed with 4% paraformaldehyde solution, permeabilized and stained with polyclonal goat anti-RSV primary antibody (Virostat) followed by donkey anti-goat IgG conjugated with Alexa Fluor 488 (AF 488) (Life technologies). Additional DAPI staining and Texas Red®-X phalloidin were performed in most assays to assess virus binding and cell shape.

### Drug treatment

Protease inhibitors were added to the cells at specific time points during the single infection cycle assay. Pre-inoculation treatment indicates a period of 1 h before inoculation during which the cells are exposed to the protease inhibitor, diluted in DMEM free of iFBS. Negative controls (NC) were incubated with DMEM free of iFBS. After 1 h, the DMEM was replaced by the inoculum, with or without (NC) inhibitor, and incubated for 2 h at 37 °C in the peri-inoculum treatment period. The inoculum was removed and replaced by DMEM with 10% iFBS with or without inhibitor and left to stand overnight for 16 h. No washing was performed between periods containing inhibitor to periods without inhibitor. For binding kinetics experiments, the peri-inoculum treatment period was performed at 4 °C for 2 h, followed by a warm DMEM change and a temperature shift to 37 °C for the remaining post-inoculation treatment period.

### Viability testing

Cells were seeded at a concentration of 200,000 cells/ml in a clear 96-well plate. They were left to adhere overnight and then treated with a 1:3 dilution series of inhibitor for 24 h. After 24 h, Resazurin was added to the wells for 4 h at 37 °C. Afterwards, the fluorescence was measured by spectrophotometry (Tecan®, GENios) to calculate the CC_50_ for AEBSF. This resulted in a CC_50_ of 0.8 mM.

### Fluorescence microscopy and image analysis

Images were obtained using a Axio Observer inverted microscope and a Compact Light Source HXP 120C with Filter set 49, 10 and 20 for blue, green and red fluorophores respectively (Zeiss) Image analysis was done using ImageJ version 2.0.0-rc-43/1.50e. Initial calculations were performed in Excel for mac version 15.18, afterwards data was transferred to Graphpad Prism 6 for further analysis.

### Statistical analysis

All experiments were performed at least in triplicate and presented as the percentage resulting from the number of RSV positive cells to the total number of cells +/− standard error of mean (SEM). Results of image counting were analyzed by a student’s t-test and ANOVA. Values *p* < 0.05 were considered statistically significant.

## Results

### Effect of different protease inhibitors on RSV infection

**Fig. 1:**
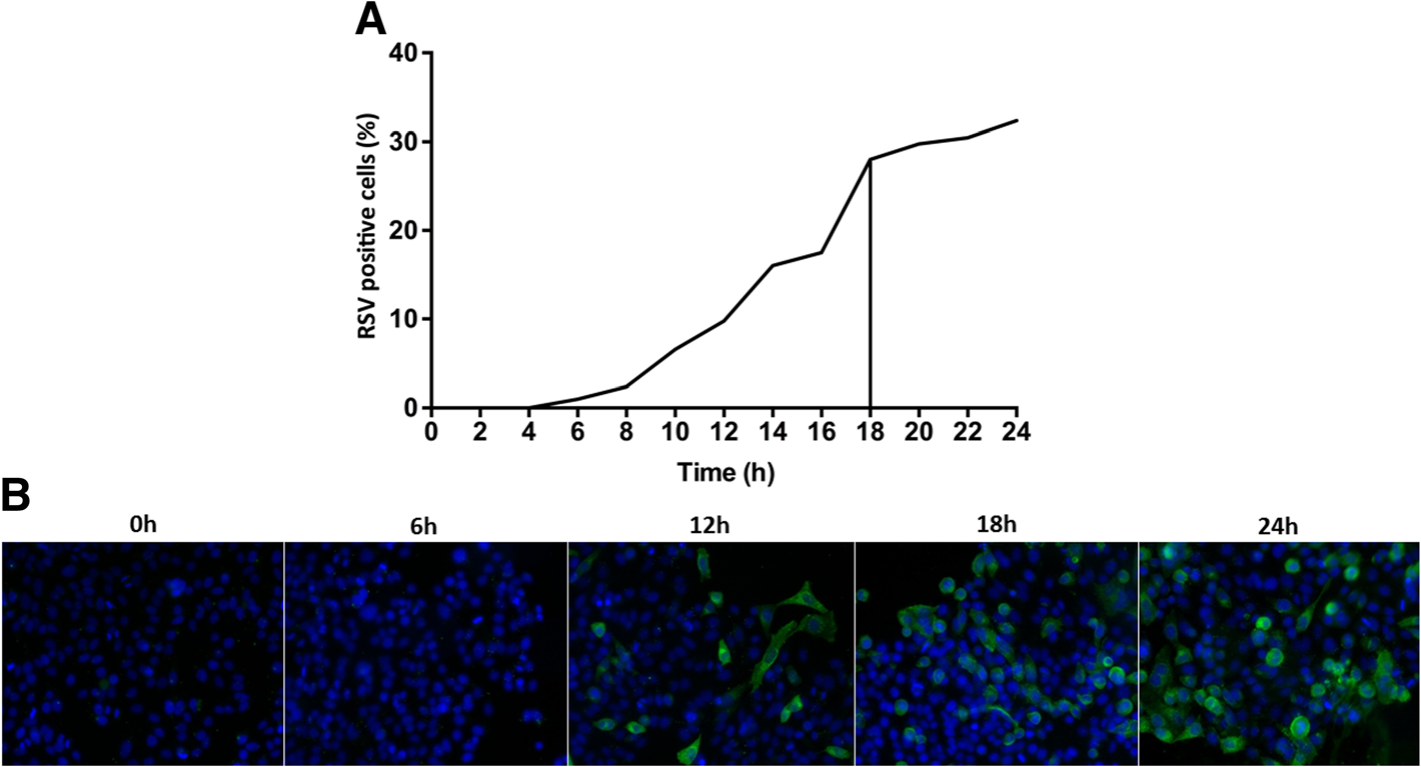
Single infection cycle. HEp-2 cells were infected with RSV A2 at 4 °C for 2 h before shifting them to 37 °C. Cells were fixed at different timepoints, permeabilized and stained with a primary antibody pAb goat anti-RSV antibody and secondary antibody Alexa fluor 488 (AF488) donkey anti-goat (IgG) (*green*). Nuclei were visualized with DAPI (*blue*). **a** Infection kinetics of RSV A2. Infected cells were counted at each indicated time point with fluorescence microscopy relative to the total number of nuclei plotted as percentage of RSV positive cells. **b** Fluorescent images (objective 20× magnification) of important timepoints during the infection kinetics

To examine the effect of protease inhibitors on RSV infection during a single replication cycle, we analyzed RSV infection in immortalized cell lines using fluorescence microscopy. This single infection cycle technique consists of an inoculation period of 2 h, followed by an incubation period to allow viral protein expression, but before a second round of replication can be detected. Afterwards, cells were fixed, stained with polyclonal antibody (pAb) anti-RSV IgG and quantified by counting the number of cells expressing RSV proteins versus the total number of nuclei. Kinetic experiments of the RSV A2 infection in cell lines allowed us to follow the replication of the virus in the cell by fluorescence microscopy (Fig. 1). Expression of viral proteins in the cytoplasm is detected by the pAb and this visualizes the viral replication (Fig. 1b): at 6 h post infection, the first signs of translation of viral proteins become visible as faint fluorescent spots in the cytoplasm. At 12 h post infection, the viral proteins have spread throughout the entire cell cytoplasm. At 18 h post infection, we reproducibly observed a plateau phase of the number of RSV positive cells, indicating all viable RSV particles have infected cells resulting in production of viral proteins (Fig. 1a). Signs of budding and release of new virus were first observed at 16 h post infection and continued throughout the rest of the assay (data not shown), indicating the start of a new infection cycle which would show new signs of infection at the earliest 6 h later. By using this single infection cycle assay, inhibitors that specifically have an effect on the entry (attachment or fusion) or intracellular replication of RSV can be identified, without interference of potential effects on virus assembly, release and spreading.

**Fig. 2:**
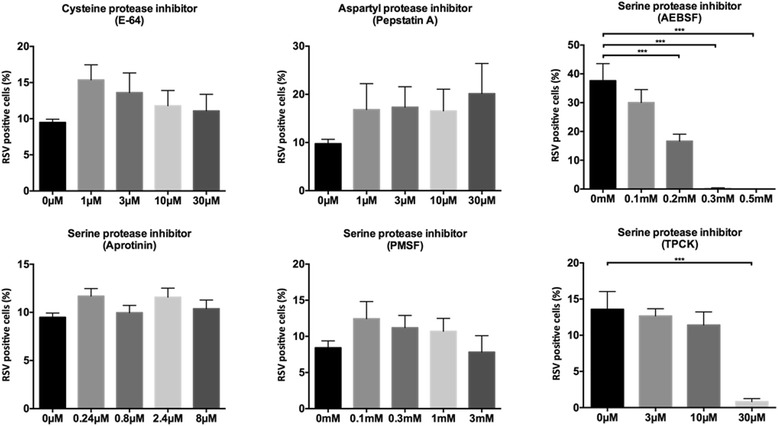
Treatment of the RSV infection with different inhibitors. HEp-2 cells were pretreated with the inhibitors in a pre-defined concentration range for 1 h, infected with RSV A2 in the presence of inhibitor for 2 h and incubated with inhibitor for an additional 16 h. Afterwards, cells were fixed, permeabilized, incubated with a pAb goat anti-RSV antibody and stained with AF488 Donkey anti-goat (IgG). Nuclei were visualized with DAPI and analyzed with fluorescence microscopy. RSV positive cells were counted and calculated to the total number of nuclei. Results are expressed as means (±SEM) (*n* = 3), significant differences compared to the untreated control (0 μM – 0 mM) are indicated by ***p < 0.001

To determine whether RSV entry and/or intracellular replication is vulnerable to inhibition of cellular proteases, we determined the impact of 3 commercially available protease inhibitors; E-64, a cysteine peptidase inhibitor, Pepstatin A, an aspartyl protease inhibitor, and AEBSF, a serine protease inhibitor (Table 1). HEp-2 cells were pre-treated with the inhibitor for 1 h, followed by a 2 h inoculation period at 37 °C in the presence of the inhibitor. Next, the medium was replaced by complete medium also containing the inhibitor. Cells were incubated for an additional 16 h at 37 °C, fixed and stained for fluorescence microscopy. Out of these 3 inhibitors, only AEBSF showed a dose dependent reduction of infection and a nearly complete block of the RSV infection at a concentration of 0.3 mM (Fig. 2).

**Table 1:** Overview of protease inhibitors used

Protease inhibitor	Characteristics	Cell permeable	Typical Working concentrations	Used working concentrations	References
E-64	Cysteine protease inhibitor that also inhibits trypsin	YES	1 – 10 μM	1 – 30 μM	Product data; [40, 41]
Pepstatin A	Aspartic protease inhibitor such as renin, chymosin and pepsin	INEFFICIENT	1 μM	1 – 30 μM	Product data; [40, 42]
AEBSF	Broad spectrum serine protease inhibitor	YES	0.1–1.0 mM	0.1–0.5 mM	Product data; [40, 43, 44]
Aprotinin	Serine protease inhibitor that does not inhibit thrombin	NO	0.3 μM	0.8 – 8 μM	Product data; [40, 45]
PMSF	Broad spectrum serine protease inhibitor	YES	0.1–1.0 mM	0.1 – 3 mM	Product data; [46, 47]
TPCK	Inhibits chymotrypsin-like serine proteases	YES	10 – 100 μM	3 – 30 μM	Product data; [48, 49]

To validate the effect of AEBSF in HEp-2 cells, the serine protease inhibitors TPCK, which mainly inhibits chymotrypsin-like proteases, aprotinin which is unable to pass the cell membrane to help distinguish between intracellular and extracellular mode of action, and PMSF which is a less stable variant of AEBSF that rapidly hydrolyses in aqueous solutions (Table 1), were tested for their capabilities to block RSV infection. Aprotinin and PMSF treatment did not result in a significant decrease in RSV infection indicating that the inhibition of AEBSF probably occurs inside the cell and requires the compound to withstand hydrolysis long enough to inhibit the key host protease. TPCK treatment also resulted in a decrease in infected cells, however only at the highest concentration tested.

### AEBSF blocks RSV infection in different cell lines

**Fig. 3:**
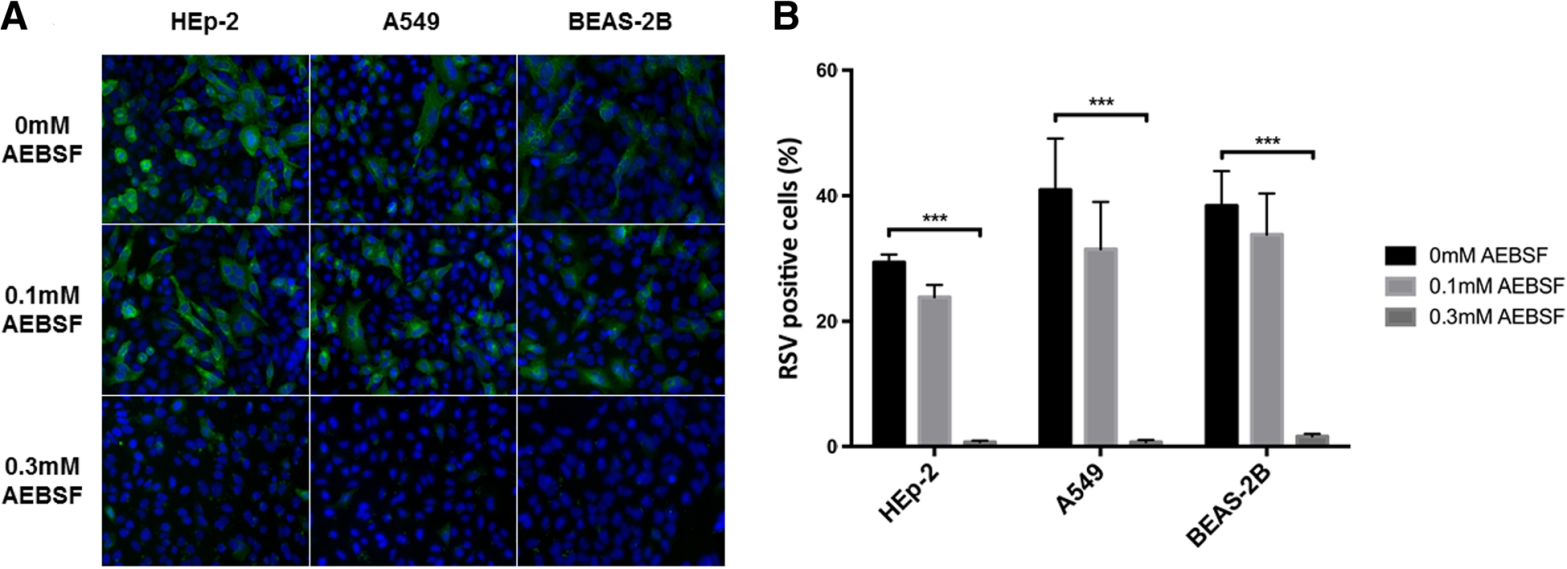
AEBSF treatment during RSV infection in different cell lines. HEp-2 cells, A549 cells and BEAS-2B cells were pretreated with AEBSF for 1 h, infected with RSV A2 in the presence of AEBSF and incubated with inhibitor for 16 h. Afterwards cells were fixed, permeabilized, incubated with a pAb goat anti-RSV antibody and stained with AF488 donkey anti-goat (IgG) (*Green*). Nuclei were visualized with DAPI (*Blue*) and analyzed with fluorescence microscopy. Infected cells were counted relative to the total number of nuclei. **a** Representative images of the treated and infected cells of each cell line. **b** Percentage of RSV positive cells after treatment with 0 mM, 0.1 mM or 0.3 mM AEBSF respectively. Data for each cell line represents means (±SEM) of 3 independent repeats, significant differences compared to the untreated control (0 mM) are indicated by ***p < 0.001

We determined whether AEBSF also inhibited RSV A2 infection in other cell lines of respiratory tract origin that are frequently used for RSV studies, such as A549 (human lung carcinoma) and BEASB-2B (transformed human bronchial epithelium) (Fig. 3). Cells were plated, pre-treated with or without AEBSF for 1 h and subjected to RSV infection for 2 h in the presence or absence of AEBSF. The inoculum was removed, and replaced with complete DMEM with or without AEBSF for the treated and control condition respectively. Cells were incubated for 16 h, followed by fixation, staining and analysis by fluorescence microscopy. A nearly complete block of the RSV infection was observed in all cell lines at a concentration of 0.3 mM AEBSF (Fig. 3b) as well as a concentration dependent effect. With 0.1 mM AEBSF RSV infection was decreased, but this was not a significant decrease. This shows that the AEBSF inhibition of RSV infection is not unique to the HEp-2 cell line but can also be observed in A549 cells as well as BEAS-2B cells.

### Early stage infection is blocked by AEBSF treatment

**Fig. 4:**
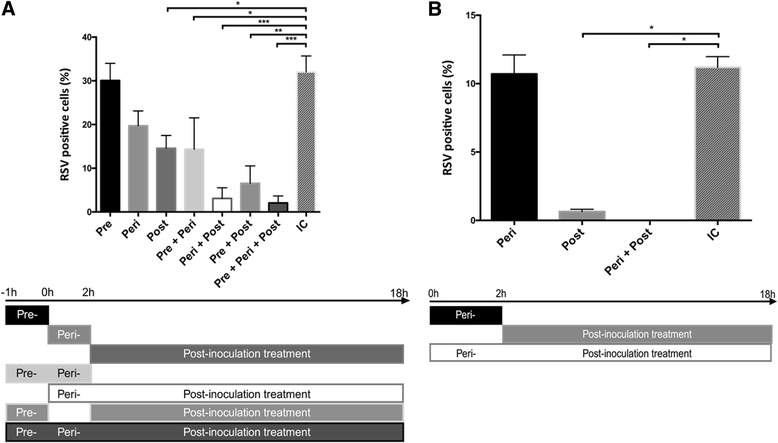
Inhibitor kinetics experiments. HEp-2 cells were treated with AEBSF, infected with RSV A2 for 2 h and incubated at 37 °C for an additional 16 h. Cells were fixed, permeabilized, incubated with a pAb goat anti-RSV antibody and stained with AF488 donkey anti-goat (IgG). Nuclei were visualized with DAPI and analyzed with fluorescence microscopy. The percentage of RSV positive cells was calculated as the number of positive cells relative to the total number of nuclei. **a** HEp-2 cells were treated with 0.3 mM AEBSF pre-, peri- or post-inoculation or combinations of these three as indicated below the graph. Data represents means (±SEM) of 3 independent repeats, significant differences compared to the untreated infection control (IC) are indicated by *p < 0.05, **p < 0.01, ***p < 0.001. **b** HEp-2 cells were treated with 0.3 mM AEBSF peri-, post- or a combined treatment of peri- and post-inoculation. Cells were incubated with virus for 2 h at 4 °C to allow attachment but not fusion. Cells were shifted to 37 °C for an additional 16 h. Results are expressed as means (±SEM) (*n* = 3), significant differences compared to the untreated infection control (IC) are indicated by *p < 0.05, **p < 0.01, ***p < 0.001

In order to determine at which stage AEBSF treatment blocks RSV A2 infection, we divided the treatment into three stages. At each stage, the medium was changed (Fig. 4a). Pre-inoculation treatment was limited to a treatment of 1 h before inoculation in DMEM without inactivated fetal bovine serum (iFBS) in order to block pre-existing active proteases. Peri-inoculation treatment was comprised of treatment during the 2 h inoculation phase which would inhibit proteases needed for attachment, fusion and uncoating. The post-inoculation phase started when the inoculum was removed 2 h post inoculation and replaced with complete medium. AEBSF was administered to the culture in a final concentration of 0.3 mM in each phase separately or in multiple phases. These time-of-addition experiments indicated that treatment only during the pre-inoculation phase did not result in a significant decrease of the RSV infection. Minor, but significant decreases were observed in the peri-inoculation only treatment, and the post-inoculation treatment only, as well as the combined treatment comprised of pre- and peri-inoculation treatment. Treatment that combines the post-inoculation phase with either the pre- or the peri-inoculation phase did result in a larger significant decrease of RSV infection. The combined peri- and post-inoculation treatment resulted in a nearly complete block that was comparable to the block observed in earlier experiments in which the treatment was comprised of pre-, peri- and post-inoculation treatment.

Given these results, we hypothesized that AEBSF needed to be present during the entry phase of RSV. In order to test this, we adapted the previous setup of the time-of-addition experiments to inoculation for 2 h at 4 °C to induce a more synchronized entry of the virion when the culture was shifted to 37 °C (Fig. 4b). Cells were placed at 4 °C 1 h prior to RSV infection to ensure a completely cooled culture. Cold inoculum was placed on the cells, followed by incubation at 4 °C for 2 h to allow attachment of the virus. Afterwards, the inoculum was removed, the cells were washed once with cold medium to remove unbound particles and complete medium at 37 °C was added to the cells to induce a temperature shift to 37 °C. Cells were further incubated at 37 °C for 18 h, fixed, stained and analyzed by fluorescence microscopy. Treatment during the peri-inoculation phase at 4 °C only did not result in a decrease in RSV infection which we considered normal since the cell metabolism is shut down at 4 °C and the virus particle only attaches at this temperature, however addition of AEBSF at the temperature change in the post-inoculation phase did result in a significant decrease of infected cells. This decrease is similar to the near complete block observed when AEBSF is present during combined peri-inoculation and post-inoculation treatment. These results confirm previous results and suggest that AEBSF treatment of RSV infection is most potent after the attachment of the virion to the host cell and before the start of replication.

### The inhibiting effect occurs in RSV A2 as well as in clinical isolates

**Fig. 5:**
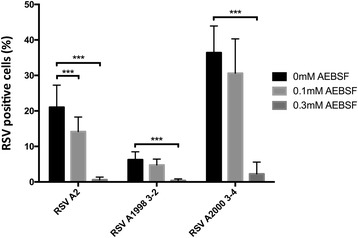
Sensitivity of clinical RSV isolates to AEBSF treatment. HEp-2 cells were infected with RSV A2, or clinical isolates RSV A1998 3–2 or RSV A2000 3–4. Cells were infected for 2 h at 37 °C in the presence of 0 mM, 0.1 mM and 0.3 mM AEBSF and further incubated for 16 h with the same concentration of inhibitor. Afterwards, Cells were fixed, permeabilized, incubated with a pAb Goat Anti-RSV antibody and stained with AF488 Donkey anti-Goat (IgG). Nuclei were visualized with DAPI and analyzed with fluorescence microscopy. The percentage of RSV positive cells was calculated as the number of positive cells relative to the total number of nuclei. Results are expressed as means (±SEM) (*n* = 3), significant differences compared to the untreated infection control (0 mM) are indicated by ***p < 0.001

To address whether our results would apply to clinical isolates as well as the RSV A2 lab strain, we tested HEp-2 cultures infected with RSV A1998 3–2 and RSV A2000 3–4 clinical isolates for their sensitivity to AEBSF treatment (Fig 5). Cells were infected for 2 h at 37 °C in the presence or absence of AEBSF. The inoculum was removed and replaced with complete DMEM with or without AEBSF in the treated and control conditions respectively. Both clinical isolates show a minor decrease in the number of RSV positive cells when treated with 0.1 mM AEBSF and a significant large decrease when treated with 0.3 mM AEBSF, indicating that inhibition is probably a general feature of RSV.

## Discussion

Respiratory syncytial virus (RSV) infection causes serious lower respiratory tract infections worldwide that affect mainly children and elderly. Despite ongoing efforts and research advances, no vaccines or therapeutics have been marketed, except for passive immunoprophylaxis, available only for high-risk infants [11]. In the vast array of possible paths toward a working therapeutic, inhibition of host proteases to affect viral infection and replication has advantages; host genes have lower mutation frequencies and therefore reduce the risk of acquiring drug resistance, which has been demonstrated for RSV with an inhibitor developed against Hsp90 [17]. Inhibiting host proteases may also provide the possibility to inhibit multiple viruses that rely on the same protease for their replication cycle, offering the potential for broad-spectrum anti-viral activity [17, 18].

Experiments performed on different myxoviruses [31] assessed the effect of several protease inhibitors on the myxovirus infection after 4 to 6 days. None were found to inhibit RSV infection whereas several inhibited influenza viruses A and B. These experiments however did not account for short term effects on the infection. Since many of the compounds evaluated may have a limited half-life in cell culture or inhibit an enzyme pool that is quickly replenished, it is important to address inhibition effects when the compound is still active. Therefore, we developed an assay to visualize and quantify one cycle of RSV infection in immortalized cell lines, 18 h after inoculation by using antibody staining to follow the protein replication of RSV. This allows for a sensitive detection of potential effects of protease inhibitors on the RSV infection. This single infection cycle assay can pick up disturbances in the replication at very early stages of the infection, which would result in a decrease of infected cells or a change in cell morphology that can be easily detected by counting and evaluating the number and shape of infected cells with fluorescence microscopy.

4-(2-Aminoethyl) benzene sulfonyl fluoride hydrochloride (AEBSF) is a broad serine protease inhibitor that inhibits proteases by reacting with the hydroxyl of the serine residu in the active site to form sulfonyl derivatives. It has been shown to also reduce reactive oxygen species without interfering with vital parameters [32–34]. In addition, AEBSF reduces airway inflammation in a food-allergen mouse model [35] and a cockroach-allergen induced mouse model [34]. Its potential to reduce airway inflammation may be an additional advantage for the treatment of RSV infection since it is well known that RSV infection results in a skewed immune response characterized by airway obstruction and inflammation [36–39].

In our experiments, we found that AEBSF and TPCK, both serine protease inhibitors, are capable of inhibiting RSV infection in the timeframe of the first infection cycle. None of the other inhibitors that were tested, consisting of inhibitors for different protease families and comparable serine protease inhibitors, were able to produce a similar blockage of RSV fusion or protein replication as detected by fluorescence microscopy.

To ensure that the inhibitory effect of AEBSF was due to inhibition of host cell factors that were not specifically linked to one cell line, we also tested A549 and BEAS-2B cell lines. In both cell lines AEBSF clearly inhibited RSV infection similarly as in HEp-2 cells. This suggests that the effect on the RSV infection by AEBSF treatment is not specific for one cell line.

For the time-of-addition experiments at 37 °C, we divided the experiment in 3 phases: a pre-treatment of 1 h, a peri-inoculation treatment of 2 h and a post-inoculation treatment of 16 h. These experiments clearly showed that treatment would only result in a near complete block when AEBSF was present at the same time as the virus inoculum. Pre-treatment did not differ significantly from the non-treated control and neither did peri-inoculation treatment at 37 °C. Post-inoculation treatment resulted in a significant decrease of the infection as did all the treatments combining phases. The most notable decrease in RSV infection was achieved by the combined treatment of peri- and post-inoculation treatment. This decrease was comparable to the earlier experiments in which the treatment was continued throughout all phases, which indicates that pre-treatment with AEBSF has no effect on the RSV infection.

These results lead us to hypothesize that it may be fusion or uncoating which is compromised, and therefore is blocking the further infection after treatment with AEBSF. However, in our experiments at 37 °C, pre-treatment of the cells had no effect on infection, indicating that the cellular protease that mediates fusion or uncoating is not yet present, active or accessible. This suggests that the binding of RSV is needed to induce enzyme activity that can be blocked by the inhibitor. This hypothesis was tested by inoculating the cells at 4 °C, at which the virus can attach to the cells but cannot fuse. As also enzymatic activity is halted at 4 °C, binding of the particle to the cell will not yet influence the activity or accessibility of the enzyme that is inhibited by AEBSF. Afterwards, the cells were given warm medium and were incubated at 37 °C to induce fusion of the virus with the host cell as well as re-activation of enzymatic processes. A near complete block of RSV infection was observed in the HEp-2 cells that were treated with AEBSF starting from the moment the cells were placed at 37 °C, compared with the marginal decrease in the cells treated peri-inoculation at 4 °C. This indicated that AEBSF is most potent at the moment of the temperature shift, presumably the time of fusion of the particle with the host cell.

Future research would have to point out the precise mechanism of action of AEBSF during the RSV infection and possible medical applications of this inhibition. Furthermore, the combined effect of AEBSF in blocking RSV infection and a possible reduction of airway inflammation, may be an additional advantage in the search towards a working therapeutic for RSV infection. In vivo experiments can enlighten the combined effect of blocking the RSV infection and suppression of the immune system which was suggested by studies in allergen models [33–35].

## Conclusion

We discovered that the RSV infection can be inhibited by a broad serine protease inhibitor, 4-(2-Aminoethyl) benzene sulfonyl fluoride hydrochloride (AEBSF). We confirmed that the effect of the inhibition is independent of the cell line or RSV isolate used. We determined the time point at which treatment with the inhibitor was most potent and found it to coincide with the expected moment of fusion of the virion with the host cell.

These results call attention to the fact that gaps still remain in our knowledge of the molecular entry process of RSV, indicating the importance to identify which proteases, that are blocked by AEBSF, are involved in RSV entry and replication. In addition, their mechanism of action needs to be elucidated*.* This additional information may help to develop new therapeutics to reduce the burden that is caused by RSV related disease in young children and elderly.

## Abbreviations

AEBSF: 4-(2-Aminoethyl) benzene sulfonyl fluoride hydrochloride
AF488: Alexa fluor 488
CPE: Cytopathic effects
DAPI: 4′,6-diamidino-2-phenylindole
DMEM: Dulbecco’s modified Eagle medium
E-64: Trans-epoxysuccinyl-L-leucylamido (4-guanido)butane
F: Fusion protein
G: Attachment glycoprotein
iFBS: Inactivated fetal bovine serum
LRTI: Lower respiratory tract infection
NC: Non-treated control
pAb: Polyclonal antibody
PepA: Pepstatin A
PFU: Plaque forming units
PMSF: Phenyl methyl sulfonyl fluoride
RSV: Respiratory syncytial virus
SEM: Standard error of mean
SH: Small hydrophobic protein
TPCK: Tosyl phenylalanyl chloromethyl ketone
